# Study protocol of the GLOW study: maximising treatment options for recurrent glioblastoma patients by whole genome sequencing-based diagnostics—a prospective multicenter cohort study

**DOI:** 10.1186/s12920-022-01343-4

**Published:** 2022-11-04

**Authors:** Mark P. van Opijnen, Marike L. D. Broekman, Filip Y. F. de Vos, Edwin Cuppen, Jacobus J. M. van der Hoeven, Myra E. van Linde, Annette Compter, Laurens V. Beerepoot, Martin J. van den Bent, Maaike J. Vos, Helle-Brit Fiebrich, Johan A. F. Koekkoek, Ann Hoeben, Kuan H. Kho, Chantal M. L. Driessen, Hanne-Rinck Jeltema, Pierre A. J. T. Robe, Sybren L. N. Maas

**Affiliations:** 1 Department of Neurosurgery, Haaglanden Medical Center, The Hague, The Netherlands; 2 Department of Neurosurgery, Leiden University Medical Center, Leiden, The Netherlands; 3 Department of Medical Oncology, Utrecht University Medical Center, Utrecht, The Netherlands; 4 Hartwig Medical Foundation, Amsterdam, The Netherlands; 5 Center for Molecular Medicine and Oncode Institute, University Medical Center Utrecht, Utrecht, The Netherlands; 6 Department of Medical Oncology, Amsterdam University Medical Centers, Amsterdam, The Netherlands; 7 Department of Neurology, Netherlands Cancer Institute-Antoni Van Leeuwenhoek, Amsterdam, The Netherlands; 8 Department of Medical Oncology, Elisabeth-Tweesteden Hospital, Tilburg, The Netherlands; 9 Department of Neurology, Erasmus Medical Center, Rotterdam, The Netherlands; 10 Department of Neurology, Haaglanden Medical Center, The Hague, The Netherlands; 11 Department of Medical Oncology, Isala Clinics, Zwolle, The Netherlands; 12 Department of Neurology, Leiden University Medical Center, Leiden, The Netherlands; 13 Department of Medical Oncology, University Hospital Maastricht, Maastricht, The Netherlands; 14 Department of Neurosurgery, Neurocenter, Medisch Spectrum Twente, Enschede, The Netherlands; 15 Clinical Neurophysiology Group, University of Twente, Enschede, The Netherlands; 16 Department of Medical Oncology, Radboud University Medical Center, Nijmegen, The Netherlands; 17 Department of Neurosurgery, University Medical Center Groningen, Groningen, The Netherlands; 18 Department of Neurology and Neurosurgery, University Medical Center Utrecht, Utrecht, The Netherlands; 19 Department of Pathology, Leiden University Medical Center, Leiden, The Netherlands; 20 Department of Pathology, University Medical Center Utrecht, Utrecht, The Netherlands

**Keywords:** Glioblastoma, Whole genome sequencing, Treatment options, Diagnostics, Recurrence

## Abstract

**Background:**

Glioblastoma (GBM), the most common glial primary brain tumour, is without exception lethal. Every year approximately 600 patients are diagnosed with this heterogeneous disease in The Netherlands. Despite neurosurgery, chemo -and radiation therapy, these tumours inevitably recur. Currently, there is no gold standard at time of recurrence and treatment options are limited. Unfortunately, the results of dedicated trials with new drugs have been very disappointing. The goal of the project is to obtain the evidence for changing standard of care (SOC) procedures to include whole genome sequencing (WGS) and consequently adapt care guidelines for this specific patient group with very poor prognosis by offering optimal and timely benefit from novel therapies, even in the absence of traditional registration trials for this small volume cancer indication.

**Methods:**

The GLOW study is a prospective diagnostic cohort study executed through collaboration of the Hartwig Medical Foundation (Hartwig, a non-profit organisation) and twelve Dutch centers that perform neurosurgery and/or treat GBM patients. A total of 200 patients with a first recurrence of a glioblastoma will be included. Dual primary endpoint is the percentage of patients who receive targeted therapy based on the WGS report and overall survival. Secondary endpoints include WGS report success rate and number of targeted treatments available based on WGS reports and number of patients starting a treatment in presence of an actionable variant. At recurrence, study participants will undergo SOC neurosurgical resection. Tumour material will then, together with a blood sample, be sent to Hartwig where it will be analysed by WGS. A diagnostic report with therapy guidance, including potential matching off-label drugs and available clinical trials will then be sent back to the treating physician for discussing of the results in molecular tumour boards and targeted treatment decision making.

**Discussion:**

The GLOW study aims to provide the scientific evidence for changing the SOC diagnostics for patients with a recurrent glioblastoma by investigating complete genome diagnostics to maximize treatment options for this patient group.

*Trial registration:* ClinicalTrials.gov Identifier: NCT05186064.

**Supplementary Information:**

The online version contains supplementary material available at 10.1186/s12920-022-01343-4.

## Background

Glioblastoma (GBM), the most common glial primary brain tumour, is almost always lethal. In the Netherlands, every year approximately 600 patients are diagnosed with this heterogeneous disease. Standard treatment for patients with newly diagnosed GBM consists of maximal safe surgical resection followed by postoperative radiation with concomitant and adjuvant temozolomide therapy [1]. Despite this intensive treatment scheme, these tumours inevitably recur and the prognosis of patients remains poor with a median survival of 14 months [2]. At the time of recurrence, only a small number of patients with well-localized tumours are eligible for re-resection. Systemic treatment is commonly suggested for recurrence, of which nitrosoureas or retreatment with temozolomide being mostly used with limited progression-free survival rates at 6 months (15–20%) and objective response rate of less than 10% [3–7]. Patients with an O6-methylguanine DNA methyltransferase (MGMT) promoter-methylated recurrent tumour may benefit from a temozolomide rechallenge, from lomustine or even the combination of both [8–10]. Outside of the European Union, bevacizumab has been approved for relapsed GBM [11, 12]. Some patients with relapsed GBM undergo re-irradiation, which may result in local disease control in a proportion of patients [13–17]. However, this is not always feasible due to the hazards of cumulative (cognitive) neurotoxicity.

Unfortunately, the results of dedicated trials with new drugs have been very disappointing. Target pre-screening, if applicable, was usually performed on archival tumour material, limited gene panels were used and not in every case a central review was performed. Targeted treatment options are becoming increasingly available for cancer patients, however studies on molecular targets for recurrent GBM patients have not yet led to clinical advantages [18]. Still, there is a major unmet need for this patient category as demonstrated by the limited treatment options and very poor survival. Furthermore, the organisation of standard-of-care (SOC) molecular testing for GBM is suboptimal. First, molecular tests are currently performed sequentially, which takes more time, especially in absence of gene panels. Second, because of this organization, tissue might become scarce. Third, different centers use different molecular panels, which are not all tailored towards identifying relevant biomarkers for (experimental) targeted treatments. Whole genome sequencing (WGS) will provide all molecular information in a single test and within a limited time of ten to fourteen days. Furthermore, additional stratification biomarkers for treatments can be identified using WGS. Although WGS is validated as a clinical diagnostic test [19, 20], its implementation in routine care environments is still slowly growing, although in the Netherlands, the non-profit organisation Hartwig provides access to WGS-based testing to all hospitals. The potential of WGS in the area of personalised medicine for patients with cancer has been demonstrated before, but it has never been prospectively studied as a SOC procedure in patients with a recurrent GBM [20, 21].

Actionability of a molecular alteration is based on information in public knowledge bases, including the Clinical Knowledgebase (CKB), Oncology Knowledge Base (OncoKB), the Clinical Interpretation of Variants in Cancer (CIViC), and can be split by evidence levels according to stablished classification levels: including the six level ESCAT classification [22]. Hypothetical target molecular alterations are those that, at minimum, are associated with preclinical evidence linking the alteration with drug activity. According to the ESCAT classification, treatment should then only be considered in the context of early clinical trials and lack of clinical data should be stressed to patients. To demonstrate that such hypothesized treatments are effective, down-stream clinical studies are required which are facilitated by effective and comprehensive identification of these molecular events without repeating past experiences with drugs that were proven to be ineffective. These trials should also investigate and link pharmacodynamics to the clinical utility of the targeted therapy, since not all drugs will effectively cross the blood–brain barrier.

The GLioblastoma targeted treatment Option maximization by Wgs (GLOW) study aims to evaluate the diagnostic value of extensive molecular diagnostics based on complete genome sequencing for patients with a first recurrence of their glioblastoma undergoing surgery for the recurrence. Consequently, this might result in the adaption of care guidelines by offering optimal and timely benefit from novel therapies, even in the absence of traditional registration trials for this small volume cancer indication.

## Objectives

### Primary objective

The primary objective of the GLOW study is to determine the percentage of patients who receive targeted therapy after surgery, including experimental therapy based on the WGS report, which should ultimately result in more effective treatment (not part of the study) and improved survival, which will be measured as overall survival (OS) within GLOW.

### Secondary objectives

There are several secondary objectives in this study. First, improvement of progression-free survival and overall survival by three months for patients that are treated based on WGS results. Second, to determine the percentage of tumour samples with sufficient quality for WGS analysis obtained during routine neurosurgical reresection. Third, to determine the percentage of tumour samples with an informative mutational profile, i.e. the number of patients with actionable mutations and number of actionable mutations per patient. Finally, to determine access to registered drugs for non-registered indications (i.e. off-label use) for these patients in The Netherlands.

## Methods/design

### Study design

**Fig. 1:**
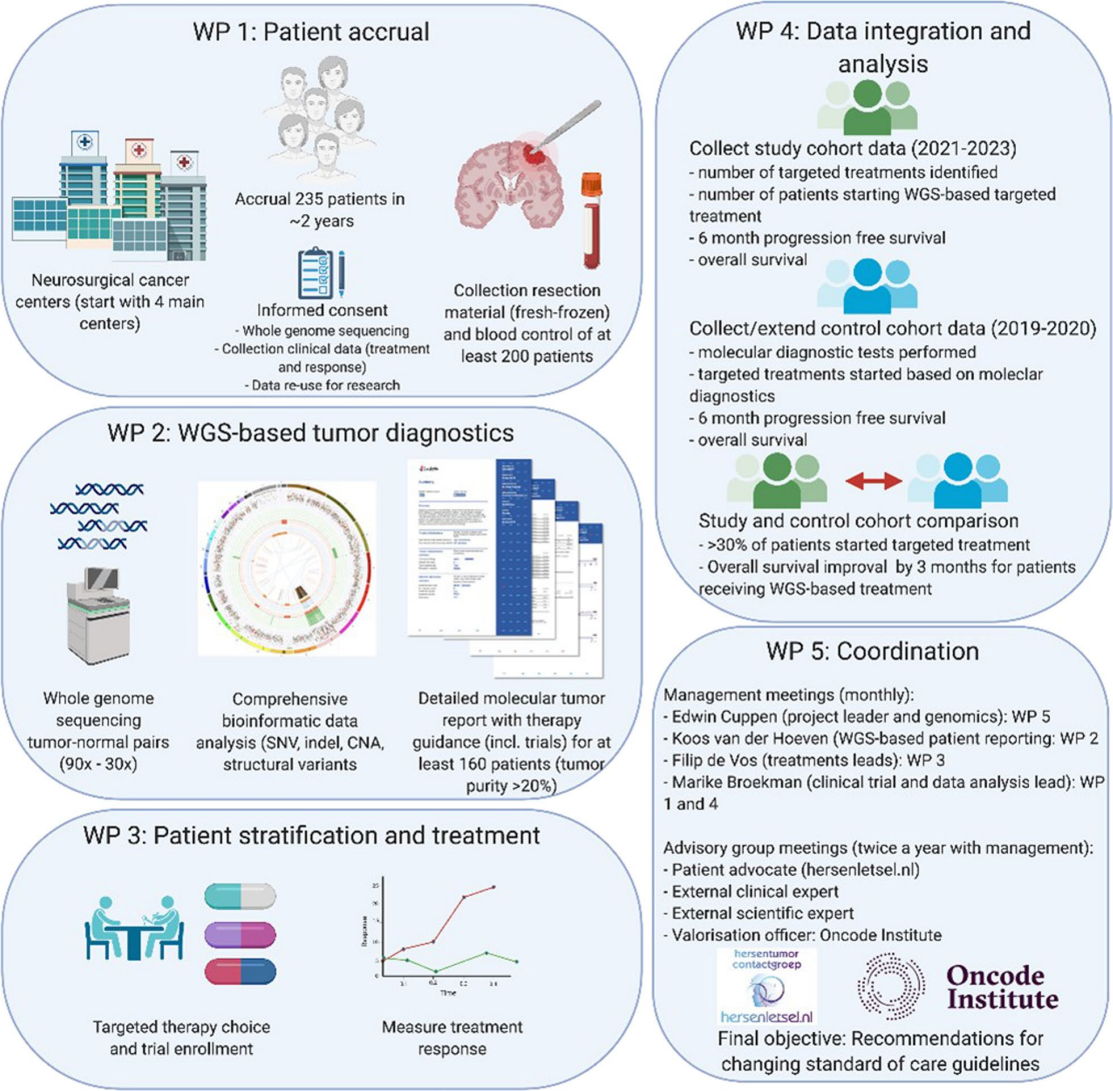
Design of the GLOW study with work packages (WP) overview

The GLOW study is a prospective diagnostic cohort study executed through collaboration of the Hartwig Medical Foundation (Hartwig, a non-profit organisation) and twelve Dutch centers that perform neurosurgery and/or treat GBM patients. The study aims to obtain, besides surgery, a more accurate pre-treatment stratification of recurrent GBM patients by obtaining fresh tumour samples and a blood sample (obtained during reresection as part of SOC) for WGS analysis leading to targeted treatment and eventual better progression free and overall survival. The patient outcomes of the prospective cohort will be compared with a similar-sized multicenter historical cohort of patients, who have not received routine WGS, seen between 2019 and 2020 in Utrecht University Medical Center (UMCU) and Haaglanden Medical Center (HMC). An independent data monitoring committee (DMC) is established to ensure independent trial supervision. The DMC will monitor the recruitment, the reported adverse events and the data quality after inclusion of the tenth patient, and at least once a year. The study design is summarised in Fig. 1. The study is registered on ClinicalTrials.gov with number NCT05186064.

### Study population

Within two years from the clinical phase, 235 patients will be recruited. Adult patients with a histopathologically confirmed isocitrate dehydrogenase (IDH) wildtype (wt) glioblastoma with a first recurrence after radiotherapy and/or systemic therapy and who are suited for SOC reresection, are eligible to participate in this study. The patients should have a life expectancy of at least three months, allowing adequate follow-up of toxicity and antitumour activity, together with a Karnofsky Performance Status (KPS) of at least seventy, since the patients should be deemed eligible for targeted treatment options, also in a clinical trial setting. Finally, the patients have to be able and willing to give written informed consent. Potential subjects who currently receive antitumour treatment will be excluded, although patients may enter other studies after WGS-based treatment decision making is completed. Patients with any other clinically significant medical condition which, in the opinion of the treating physician, makes it undesirable for the patient to participate in medication studies or which could jeopardize compliance with study requirements including, but not limited to, ongoing or active infection, significant uncontrolled hypertension, or severe psychiatric illness/social situations, will be excluded as well.

### Statistical analysis

There are no formal statistical considerations that underlie this study as the study assesses the impact of using WGS in diagnostics versus current standard of care and patients will receive potentially a broad range of treatments with variable outcome expectations. First interim analysis of the results, on which premature termination or modification of the study will be based, will be started when the clinical follow-up data of 100 WGS analysed patients is available.

### Sample size calculation

The aim is to include a total of 235 patients in this study. Based on clinical expertise, around 15% of the initially included patients are expected to not be able to undergo the planned reresection because of medical conditions or personal choices, resulting in a total of 200 patients who will be included in the GLOW study. Based on previous experience, for about 20% of patients the obtained material is unfortunately not suited for WGS due to insufficient harvest of tumour cells. Collecting procedures aimed for avoiding necrotic and low tumour purity regions and prioritizing the best suited material for molecular diagnostics should minimise this rate. Over the complete project, on average a maximum of 20% of samples will be expected to drop out due to insufficient quality for WGS, mainly due too low tumour purity. This means that a WGS based patient report will be generated for a minimum of 160 patients.

### Sample collection and processing

Study participants will undergo standard reresection of the tumour by the neurosurgeon as part of SOC. The collection of fresh frozen material will be done according to the standard operation protocol. Upon tissue collection, multiple samples will be sent to the pathology department of the neurosurgical center. After confirmation of the diagnosis recurrent glioblastoma, samples including information regarding the tumour cell percentage will be shipped to Hartwig for processing. Although the aim is to use 200 ng of DNA as input for WGS, all tumour samples with a minimum of 50 ng of DNA will be processed. Although not used in this study, RNA will simultaneously be isolated from the same tumour tissue and biobanked for later usage like whole transcriptome sequencing. In addition, a 10 mL blood sample will be collected from the patients to isolate normal germline DNA (i.e., not only from the tumour) in order to be able to discriminate somatic mutations from the patient’s germline DNA background variations. After diagnostic procedures by Hartwig, the samples will be stored in the local biobanks of the corresponding centers.

### DNA sequencing

Only tumours with at least 20% tumour purity will be further processed for deep sequencing by WGS. The tumour purity will be maximised by collecting multiple samples from different regions of the tumour to avoid radionecrotic samples. WGS of the tumour DNA will be performed according to the previously described standard procedures [21]. Samples with the required tumour purity will be deep-sequenced on Illumina Novaseq to an average depth of 90–100 × and the blood control samples to a depth of 30-35x. Thus, a total of four ‘standard 30x’ genome equivalents are generated per patient to be able to filter for abundantly present germline variants and to deal with tumour heterogeneity and presence of non-tumour cells in the tumour sample. This enables the reporting of somatic variants and therapeutically actionable mutations. Hartwig has established procedures for WGS under ISO17025 accreditation and the WGS-based test is already used in routine diagnostics for other indications (e.g. Cancer of Unknown Primary) and in various hospitals in The Netherlands.

### Treatment decision

The WGS report that will be made available by Hartwig (see Additional file 1 for an example) will be sent to the local pathologist and local study coordinator, who will add the report to the electronic patient files and enters relevant information to a nationwide network and registry of histo- and cytopathology in The Netherlands (PALGA) [23]. In addition, patient reports will be returned to the treating medical specialist as well as to central and local principal investigators. The neuro-oncology team will discuss the results and allocate subsequent treatment accordingly. If needed, the local neuro-oncologist can consult a centralized molecular tumour board which will also receive the anonymised report for central data management. In case of a persistent discordance between the results of WGS and SOC diagnostics, the SOC findings will be leading in the treatment decision. Such discrepancies will be followed up with revalidation of the results (e.g. to exclude sample heterogeneity as a cause) including the use of an independent orthogonal assay when needed.

### Ethical considerations

Every patient will be extensively informed about the study goals and (potential) patient impact by a local research nurse, nurse practitioner or clinical specialist, and will have to sign an informed consent before participating in the study. Potential study participants will get one to two weeks, the time between planning surgery and the operation date, to decide on participating and will get the opportunity to ask additional questions or consult the independent expert of the study. Apart from consenting to the collecting, storage and use of their tumour and blood material, the patients will be asked for their consent to being informed about relevant inherited findings in germline DNA and, if so, under which conditions. Participants can limit this choice to disease that are preventable or treatable and can provide their preference for family to obtain access to heritable information after being deceased. This germline consenting model is optimized based on patient preferences [24] and also was applied in the CPCT-02 (open, NCT01855477), WIDE (closed) [25] and DRUP (open, NCT02925234) studies. All adverse events (AEs) reported spontaneously by the subject or observed by the investigator or his staff will be recorded. All AEs will be followed until they have abated, or until a stable situation has been reached. Depending on the event, follow-up may require additional tests or medical procedures as indicated.

### Primary endpoints

Dual primary endpoint is the percentage of patients who receive targeted therapy based on the WGS report and OS. The OS of these patients will be compared to the OS of patients in the historical cohort, who have not had WGS-based treatment, and should be improved by three months at least.

### Secondary endpoints

#### Tissue collection and reports

The aim is that at least in 85% of all patients included tumour and blood collection will be successful. Feasibility of routine WGS analysis in this patient population will be measured by the percentage of patients for whom a successful WGS report can be generated. The aim is that at least 80% of the patients for which tumour and blood material was collected will receive a WGS report. Reasons for not being able to produce a patient report based on WGS include low or no tumour cellularity of the available tumour material (expected 15 to 20% based on previous experiences), low DNA yield or quality (e.g. due to necrosis, < 3%), and technical failures (< 2%).

#### Targeted treatment options

Another important endpoint is the added value of WGS indicated by the number of targeted treatment options identified. As mentioned before, actionability is based on information in public knowledge bases and can be split by ESCAT classification evidence levels [22]. Because the ESCAT levels are not yet available in public knowledge bases, Food and Drug Administration (FDA) approved drugs and drugs for which a trial is currently available, bases on the JAX CKB clinical knowledgebase, will be reported by Hartwig. Interpretation of the genomic variants in terms of pathogenicity and actionability will be done by using criteria for classifying pathogenic variants [26] and expert interpretation in molecular tumour boards.

The expectation is that at least one potentially actionable DNA alteration should be identified in at least 75% of the patients with a WGS report. Consequently, the number of experimental treatments available for these patients with a recurrent GBM will be measured. At least 50% of the identified indications should be available (albeit off-label drugs) through a study, including the DRUP study. A third endpoint regarding targeted treatment options is a doubling of the number of patients starting a targeted treatment in presence of one or more actionable variants (i.e. from 16 to 32%). We aim to dissect this increase for improvements due to diagnostics and/or availability of novel drugs by both comparing historic diagnostic yields as well as treatments given and outcomes.

#### Progression free survival

Finally, data about the median progression free survival after reresection will be collected by calculating the time between the date of the reresection and the date of clinical and/or radiological progression. The aim of the GLOW study is to improve the median progression free survival by at least three months for the patients who are treated based on WGS results compared to patients in the historical cohort who are not treated based on WGS results.

## Discussion

The GLOW study is a unique trial since it is the first time that patients with a recurrent glioblastoma will prospectively obtain a standard-WGS analysis to identify targeted treatment options that could help treatment decision after reresection. The prognosis in this patient population remains very poor, and several questions about the best treatment strategy at the time of first recurrence of the tumour are still unanswered. This study aims to generate evidence for the added value of WGS as a routine diagnostic in this patient population. If a significant benefit is demonstrated, this will show cost effectiveness. However, it is important to be aware of the limitations of this study.

From a patient’s perspective, it can be essential to know everything is done to give them an opportunity of a targeted treatment, whether experimental or not. Notwithstanding, it is crucial to remember that the GLOW study will not investigate the treatments itself, but focusses on the clinical effect of a different diagnostic strategy. We do fully realise that with today’s knowledge and available drugs, this study may not reach successful endpoints due to limited effectiveness of the mostly experimental treatments that will be given based on WGS. Secondary endpoints, as the feasibility of routine WGS diagnostics, are therefore also important for determining next steps as the future targeted drug portfolio is likely to be expanded significantly [27, 28]. Another potential limitation could be the situation in which an actionable target is found in absence of a recruiting drug study. However, previous studies on WGS-based diagnostics in cancer, i.e. the beforementioned CPCT-02 and WIDE studies, do not support this potential objection. Moreover, experimental targets will not be reported to avoid these situations. At the same time, a close monitoring of the expanded use of existing anticancer drugs could lead to new treatments [29]. Finally, the heterogeneity of glioblastoma, tumour penetrating issues and pathway redundancy are all limitations that could hamper successful targeted treatments and should therefore be kept in mind when analysing the results of this study.

In conclusion, the GLOW study aims to investigate the feasibility, validity, utility and value of WGS for recurrent GBM patients. This will allow for disclosure of potentially novel targets for therapy for these patients.

## Supplementary Information

**Additional file 1. **DNA analysis report.

## Data Availability

The datasets obtained during the current study, data management procedures or the full protocol will be available from the corresponding author upon reasonable request. Genomics data and certain clinical data of patients that have given consent for re-use of their data are made readily available through the standard controlled access mechanism of the Hartwig Medical Foundation (see https://www.hartwigmedicalfoundation.nl/applying-for-data/ for details and application forms).
