# Supplementary material for: Study protocol of the GLOW study: maximising treatment options for recurrent glioblastoma patients by whole genome sequencing-based diagnostics—a prospective multicenter cohort study
Source: BMC Med Genomics. 2022 Nov 4;15:233. doi: 10.1186/s12920-022-01343-4 (PMC9636658; doi:10.1186/s12920-022-01343-4)
Supplement: Supplementary file 1 — Additional file 1. DNA analysis report. [file 12920_2022_1343_MOESM1_ESM.pdf]

Hartwig Medical Foundation

Hartwig Medical OncoAct

DNA Analysis Report

Summary (1/2)

HMF SAMPLE ID
PNT00012345T

REPORT DATE
10-Dec-2021

HOSPITAL
HMF Testing Center

PRIMARY TUMOR LOCATION
Skin

BIOPSY LOCATION
Skin

PRIMARY TUMOR TYPE
Melanoma

The information regarding 'primary tumor location', 'primary tumor type' and 'biopsy location' is based on information received from the originating hospital.

Clinical Conclusion

Melanoma sample showing:
- activating BRAF mutation that is associated with response to BRAF-inhibitors (in combination with a MEK-inhibitor)
- complete inactivation of CDKN2A, indicating potential benefit of CDK4/6 inhibitors
- complete inactivation/loss of PTEN likely resulting in an activation of the PI3K-AKT-mTOR pathway and indicating potential benefit of mTOR/PI3K inhibitors
- high mutational burden (mutational load (ML) of 180, tumor mutation burden (TMB) of 13.6) that is potentially associated with an increased response rate to checkpoint inhibitor immunotherapy

Treatment options (tumor-type specific)

Number of alterations with therapy indication	2 | 7 (A, B) treatment(s)
Number of alterations with clinical trial eligibility	3 | 7 trial(s)

Tumor characteristics

Tumor purity	100%
Molecular tissue of origin prediction	Melanoma (likelihood=99.6%)
Tumor mutational load	High (189 mut/genome)
Microsatellite (in)stability	Stable (0.12)
HR Status	Proficient (0)
Integrated Virus	NONE

Genomic alterations in cancer genes

Genes with driver mutation	TERT, CDKN2A, BRAF
Number of reported variants	5
Amplified gene(s)	NONE
Deleted gene(s)	PTEN
Homozygously disrupted genes	NONE
Gene fusions	NONE

HMF-FOR-080 v7.24

DNA Analysis Report

Summary (2/2)

HMF SAMPLE ID
PNT00012345T

REPORT DATE
10-Dec-2021

HOSPITAL
HMF Testing Center

Pharmacogenetics

Genes with haplotypes	DPYD
Number of reported haplotypes	1
Functions of the haplotypes	Normal Function

Therapy details (Tumor type specific)

Tumor type specific evidence

TREATMENT	LEVEL	RESPONSE	GENOMIC EVENT	EVIDENCE LINKS
Cobimetinib + Vemurafenib	A	▲	BRAF p.V600E	
Dabrafenib	A	▲	BRAF p.V600E	
Dabrafenib + Trametinib	A	▲	BRAF p.V600E	1
Trametinib	A	▲	BRAF p.V600E	
Vemurafenib	A	▲	BRAF p.V600E	1
Buparlisib + Carboplatin + Paclitaxel	B	▲	PTEN partial loss	1
RO4987655	B	▲	BRAF p.V600E	1

Tumor type specific clinical trials (NL)

TRIAL	GENOMIC EVENT
Array 818-103	BRAF p.V600E
BASKET OF BASKETS (VHIO17002)	High tumor mutation load
CLXH254C12201	BRAF p.V600E
COWBOY	BRAF p.V600E
DRUP	BRAF p.V600E<br>High tumor mutation load<br>PTEN partial loss
EBIN (EORTC-1612-MG)	BRAF p.V600E
KEYNOTE-158	High tumor mutation load

Potential eligibility for DRUP is dependent on tumor type details therefore certain tumor types may not be eligible for the DRUP.

The iClusion knowledgebase is used to annotate DNA aberrations for potential clinical study eligibility. Please note clinical study eligibility depends on multiple patient and tumor characteristics of which only the DNA aberrations are considered in this report.

The Clinical Knowledgebase (CKB) is used to annotate variants of all types with clinical evidence. Only treatment associated evidence with evidence levels ((A FDA approved therapy and/or guidelines; B late clinical trials; C early clinical trials) can be reported. Potential evidence items with evidence level (D case reports and preclinical evidence) are not reported.

The symbol (▲) means that the evidence is responsive. The symbol (▼) means that the evidence is resistant. The abbreviation (P mentioned after the level of evidence) indicates the evidence is predicted responsive/resistent. More details about CKB can be found in their Glossary Of Terms

Therapy details (Other tumor types)

Evidence on other tumor types

TREATMENT	LEVEL	RESPONSE	GENOMIC EVENT	EVIDENCE LINKS
Anti-EGFR monoclonal antibody	B	▼	PTEN partial loss	1, 2
Bevacizumab	B	▼	BRAF p.V600E	1, 2
CI-1040	B	▲	BRAF p.V600E	1, 2
Cetuximab	B	▼	BRAF p.V600E	1, 2, 3, 4, 5, 6
	B	▼	PTEN partial loss	1
Cetuximab + Irinotecan + Vemurafenib	B	▲	BRAF p.V600E	1
Everolimus	B	▼	PTEN partial loss	1
Fluorouracil	B	▼	BRAF p.V600E	1
Irinotecan	B	▼	BRAF p.V600E	1
Lapatinib + Trastuzumab	B	▼	PTEN partial loss	1
Oxaliplatin	B	▼	BRAF p.V600E	1
Panitumumab	B	▼	BRAF p.V600E	1, 2, 3, 4
Selumetinib	B	▲	BRAF p.V600E	1
Sorafenib	B	▲	BRAF p.V600E	1, 2
Trastuzumab	B	▼	PTEN partial loss	1, 2
Vemurafenib	B	▼	BRAF p.V600E	1

The Clinical Knowledgebase (CKB) is used to annotate variants of all types with clinical evidence. Only treatment associated evidence with evidence levels ((A FDA approved therapy and/or guidelines; B late clinical trials; C early clinical trials) can be reported. Potential evidence items with evidence level (D case reports and preclinical evidence) are not reported.

The symbol (▲) means that the evidence is responsive. The symbol (▼) means that the evidence is resistant. The abbreviation (P mentioned after the level of evidence) indicates the evidence is predicted responsive/resistent. More details about CKB can be found in their Glossary Of Terms

Genomic alteration details (1/2)

Tumor purity & ploidy

Tumor purity	100%
Average tumor ploidy	3.1

Tumor specific variants

GENE	POSITION	VARIANT	PROTEIN	READ DEPTH	COPIES	TVAF	BIALLELIC	HOTSPOT	DRIVER
BRAF	7:140453136	c.1799T>A	p.Val600Glu	**150** / 221	6	68%	No	Yes	High
CDKN2A	9:21971153	c.203_204delCG	p.Ala68fs	**99** / 99	2	100%	Yes	Near	High
TERT	5:1295228	c.-125_-124delCCinsTT		**56** / 65	2	87%	Yes	Yes	High
SF3B1	2:198266779	c.2153C>T	p.Pro718Leu	**74** / 111	3	67%	No		Low
TP63	3:189604330	c.1497G>T	p.Met499Ile	**47** / 112	4	42%	No		Low

Tumor specific gains & losses

CHROMOSOME	REGION	GENE	TYPE	MIN COPIES	MAX COPIES	CHROMOSOME ARM COPIES
10	q23.31	PTEN	partial loss	0	2	2

Tumor specific gene fusions

NONE

Tumor specific homozygous disruptions

NONE

Tumor specific gene disruptions

LOCATION	GENE	DISRUPTED RANGE	TYPE	DISRUPTED COPIES	UNDISRUPTED COPIES
10q23.31	PTEN	Intron 5 -> Intron 6	DEL	2	0

Tumor specific viral insertions

NONE

Genomic alteration details (2/2)

Pharmacogenetics

GENE	GENOTYPE	FUNCTION	LINKED DRUGS	SOURCE
DPYD	*1_HOM	Normal Function	5-Fluorouracil;Capecitabine;Tegafur	PHARMGKB

Tumor characteristics (1/2)

HR-Deficiency score

Proficient 0

The HR-deficiency score is determined by CHORD, a WGS signature-based classifier comparing the signature of this sample with signatures found across samples with known BRCA1/BRCA2 inactivation.
Tumors with a score greater or equal than 0.5 are considered HR deficient by complete BRCA inactivation.

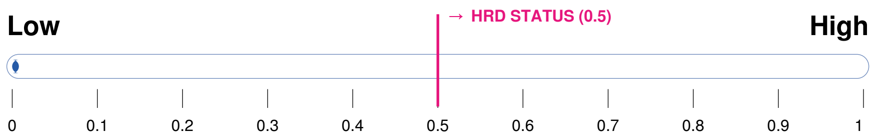

Microsatellite status

Stable 0.12

The microsatellite stability score represents the number of somatic inserts and deletes in (short) repeat sections across the whole genome of the tumor per Mb. This metric can be considered as a good marker for instability in microsatellite repeat regions. Tumors with a score greater than 4.0 are considered microsatellite unstable (MSI).

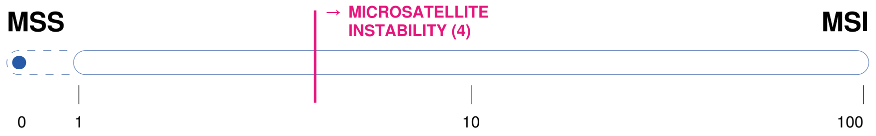

Tumor mutational load

High 189

The tumor mutational load represents the total number of somatic missense variants across the whole genome of the tumor. Patients with a mutational load over 140 could be eligible for immunotherapy within the DRUP study.

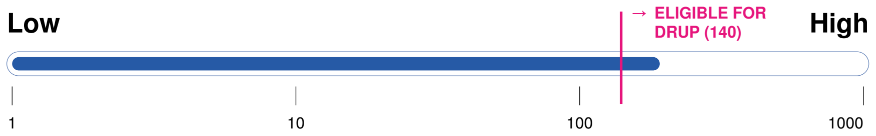

Tumor mutational burden

13.7 variants per Mb

The tumor mutational burden score represents the number of all somatic variants across the whole genome of the tumor per Mb.

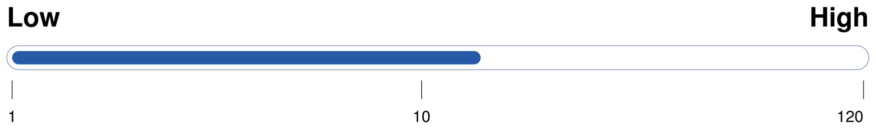

Tumor characteristics (2/2)

Molecular tissue of origin prediction

Molecular tissue of origin - Melanoma (likelihood=99.7%)

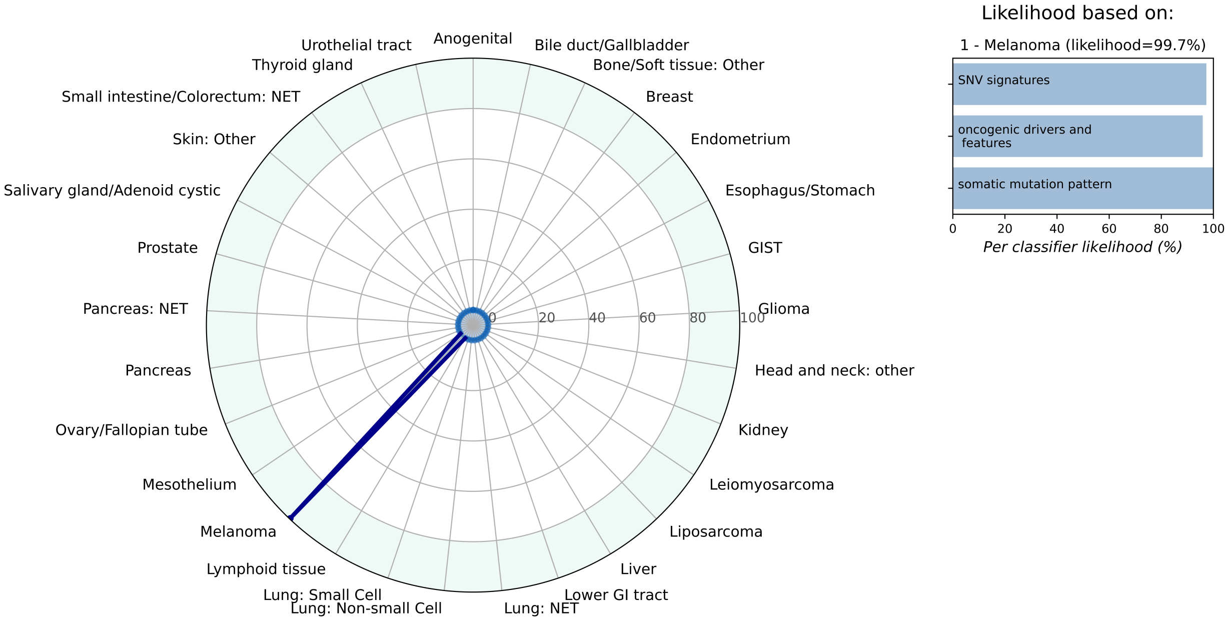

The title shows the conclusion of the prediction of the molecular tissue of origin. If none of the similarity predictions has a likelihood ≥80%, no reliable conclusion can be drawn (‘results inconclusive’).

The left plot shows the likelihoods (similarity) for all the origin types analyzed by the molecular tissue of origin prediction tool. Only when the likelihood is ≥80% (a peak in the green outer band of the plot), a reliable prediction (with >95% accuracy) can be drawn. Lower likelihoods (<80%) suggest there is similarity with that tissue of origin, but this is less strong and there is lower confidence.

The right plot(s) shows the breakdown of the strongest predicted likelihood(s) into the contribution of the 1) SNV types (related to those used in Cosmic signatures), 2) driver landscape and passenger characteristics (e.g. tumor-type specific drivers), and 3) somatic mutation pattern (mutation distribution across the genome).

CIRCOS plot

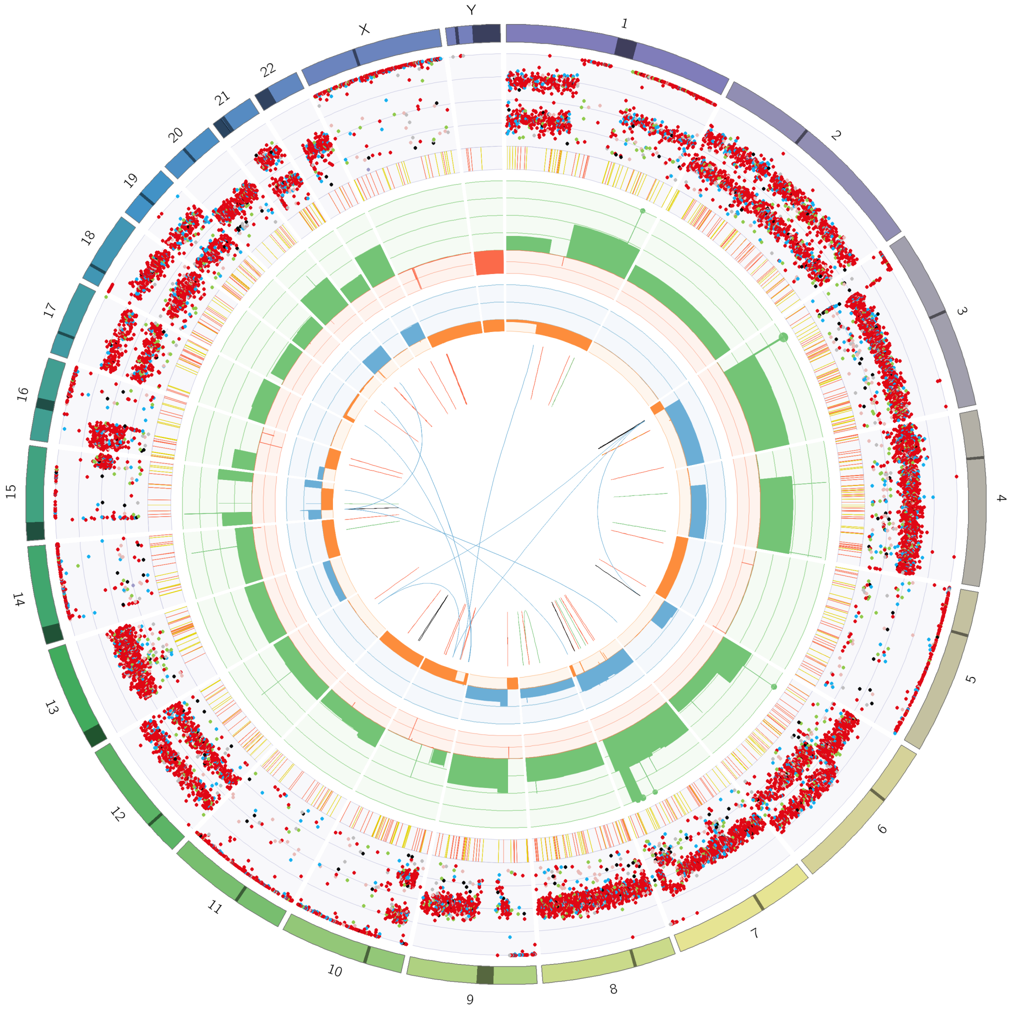

The outer first circle shows the chromosomes. The darker shaded areas represent large gaps in the human reference genome: i.e. regions of centromeres, heterochromatin & missing short arms.

The second circle shows all tumor specific variants (incl. exon, intron and intergenic regions) and are divided into an outer ring of single nucleotide polymorphism (SNP) allele frequencies and an inner ring of short insertion/deletion (INDEL) locations. Variant allele frequencies have been corrected for tumor purity and scale from 0 to 100%. Each dot represents a single variant and are colored according to the type of base change (e.g. C>T/G>A in red) and are in concordance with the coloring used in Alexandrov et al. 2013 Nature paper that describes the use of mutational signatures. INDELs are colored yellow and red for insertions and deletions respectively.

The third circle shows all observed tumor purity adjusted copy number changes, including both focal and chromosomal events. Copy number losses are indicated in red, green shows regions of copy number gain. The scale ranges from 0 (complete loss) to 6 (high level gains). If the absolute copy number is > 6 it is shown as 6 with a green dot on the diagram.

The fourth circle represents the observed 'minor allele copy numbers' across the chromosome. The range of the chart is from 0 to 3. The expected normal minor allele copy number is 1, and anything below 1 is shown as a loss and represents a LOH event (orange). Minor allele copy numbers above 1 indicate amplification events of both A and B alleles at the indicated locations (blue).

The innermost circle displays the observed structural variants within or between the chromosomes. Translocations are indicated in blue, deletions in red, insertions in yellow, tandem duplications in green and inversions in black.

Report explanation (1/2)

Details on the report in general

The analysis is based on reference genome version GRCh37.

Transcripts used for reporting can be found on https://resources.hartwigmedicalfoundation.nl in directory 'Patient-Reporting' and are generally the canonical transcripts as defined by Ensembl.

Variant detection in samples with lower tumor content is less sensitive. In case of a low tumor purity (below 20%) likelihood of failing to detect potential variants increases.

The (implied) tumor purity is the percentage of tumor cells in the tumor material based on analysis of whole genome data.

Details on the reported clinical evidence

The Clinical Knowledgebase (CKB) (https://ckbhome.jax.org/) is used to annotate variants of all types with clinical evidence, with a hyperlink to the specific evidence items when available. The evidence is gathered from CKB without further checks or interpretation. This also means that if a certain evidence item or drug-biomarker is missing from the knowledgebase it will also not be included in this report.
More details about CKB can be found in their Glossary Of Terms.
(https://ckbhome.jax.org/about/glossaryOfTerms)

Clinical trials are matched against the iClusion database https://iclusion.org including a link to the specific trial.

Hartwig Medical Foundation is not responsible for the content of the knowledgebases used to generate this report. Furthermore, Hartwig Medical Foundation is not liable and cannot be held accountable for any incorrectness, incompleteness or error of any other kind in the knowledgebases, or the external software used to harmonize and curate the knowledgebases.

Details on reported somatic variants

The 'Read Depth' displays the raw number of reads supporting the variant versus the total number of reads on the mutated position.

The 'Copies' field indicates the number of alleles present in the tumor on this particular mutated position.

The 'tVAF' field displays the variant allele frequency corrected for tumor purity.

The 'Biallelic' field indicates whether the variant is present across all alleles in the tumor (and is including variants with loss-of-heterozygosity).

The 'Driver' field is based on the driver probability calculated based on the HMF database. A variant in a gene with High driver likelihood is likely to be positively selected for during the oncogenic process.

Details on reported gene copy numbers

The lowest copy number value along the exonic regions of the canonical transcript is determined as a measure for the gene's copy number.

Copy numbers are corrected for the implied tumor purity and represent the number of copies in the tumor DNA.

Any gene with less than 0.5 copies along the entire canonical transcript is reported as a full loss.

Any gene where only a part along the canonical transcript has less than 0.5 copies is reported as a partial loss.

Any gene with more copies than 3 times the average tumor ploidy along the entire canonical transcript is reporte as a full gain.

Any gene where only a part of the canonical transcript has more copies than 3 times the average tumor ploidy is reported as a partial gain.

Details on reported gene fusions

The canonical, or otherwise longest transcript validly fused is reported.

Fusions are restricted to those in the HMF known fusion list and can be found on https://resources.hartwigmedicalfoundation.nl in directory 'Patient-Reporting'.

We additionally select fusions where one partner is promiscuous in either 5' or 3' position.

The 'Driver' field is set to HIGH in case the fusion is a known pathogenic fusion, or otherwise a fusion where the promiscuous partner is fused in an exon range that is typically observed in literature.
All other fusions get assigned a LOW driver likelihood.

Details on reported gene disruptions

Genes are reported as being disrupted if their canonical transcript has been disrupted.

The range of the disruption is indicated by the intron/exon/promoter region of the break point and the direction the disruption faces.

The type of disruption can be INV (inversion), DEL (deletion), DUP (duplication), INS (insertion), SGL (single) or BND (translocation).

A gene for which no wild type exists anymore in the tumor DNA due to disruption(s) is reported in a separate section called 'homozygous disruptions'.

Report explanation (2/2)

Details on reported viral insertions

Virusses will be reported if they are present in our reporting database as clinically relevant (HPV, MCV, HBV, EBV and HHV-8) and DNA integration for the virus can be detected. If the virus is clinically relevant and no DNA integration is found, the following conditions must be met:
- Percentage covered of the viral genome is >90%
- Coverage of the virus is higher than the expected clonal mean coverage

Reporting of EBV is independent of tumor integration. This means that to be reportable, the viral EBV genome must be covered >90% and the coverage of the virus must be higher than the expected clonal mean coverage.

Details on reported pharmacogenetics

See the directory 'Patient Reporting' in https://resources.hartwigmedicalfoundation.nl for details on the panel and for more links to advice on treatment adjustments.

The called haplotypes for a gene are the simplest combination of haplotypes that perfectly explains all of the observed variants for that gene. If no combination of haplotypes in the panel can perfectly explain the observed variants, then 'Unresolved Haplotype' is called.

Wild type is assumed when no variants are observed.

Sample details & disclaimers (1/2)

Sample details

The samples have been sequenced at **Hartwig Medical Foundation, Science Park 408, 1098XH Amsterdam**

The samples have been analyzed by Next Generation Sequencing using Whole Genome Sequencing

The HMF sample ID is: **PNT00012345T**

The germline reporting choice of this patient is: **no reporting**

The results in this report have been obtained between **01-Oct-2020** and **10-Dec-2021**

This experiment is performed on the tumor sample which arrived on **05-Oct-2020** with internal tumor barcode **FR12345678**

This experiment is performed on the blood sample which arrived on **01-Oct-2020** with internal blood barcode **FR12123488**

The results stated in this report are based on the tested tumor and blood sample.

This experiment is performed according to lab procedures: **PREP013V23-QC037V20-SEQ008V25**

This report was generated by Lieke Schoenmaker (trained IT employee) and checked by a trained Clinical Molecular Biologist in Pathology (KMBP)

This report is addressed to: **PI, HMF Testing Center, 1000 AB AMSTERDAM**

Comments: This is a test report and is based on COLO829. Where is referred to CKB, VICC evidence is listed due to licensing restrictions.

Disclaimer

The data on which this report is based is generated from tests that are performed under ISO/ICE-17025:2017 TESTING L633 accreditation and have passed all internal quality controls.

This report is generated by patient reporter **version 7.24** based on **HMF-FOR-080.**

UDI-DI: **(01) 8720299486010(8012)v5.25.**

The OncoAct user manual can be found at https://www.oncoact.nl/manual.

This report is based on pipeline version **5.25.**

The 'primary tumor location' and 'primary tumor type' have influence on the clinical evidence/study matching. No check is performed to verify the received information.

The conclusion of this report is based solely on the results of the DNA sequencing of the tumor and the received tumor type. Final interpretation of the clinical consequence of this report should therefore always be performed by the treating physician.

Based on a tumor purity of at least 20%, the test has a sensitivity of >95% for detection of somatic variants and >95% for detection of translocations and gene copy number changes.

For feedback or complaints please contact **qualitysystem@hartwigmedicalfoundation.nl.**

For questions about the contents of this report, please contact **diagnosticssupport@hartwigmedicalfoundation.nl.**

RVA

Edwin Cuppen,

Director Hartwig Medical Foundation

Sample details & disclaimers (2/2)

— End of report —
